# Effects of blood flow restriction exercise interventions on patellofemoral pain syndrome: a systematic review and meta-analysis

**DOI:** 10.3389/fphys.2026.1859305

**Published:** 2026-06-18

**Authors:** Chunwei Hou, Zewei Liu, Chunhui Wang, Jingshuo Wang, Haitao Zhou, Xiaolong Ma, Shaoqi Huang, Jingqi Tao, Zheyu Xiong

**Affiliations:** 1 Shijiazhuang Campus, Army Engineering University of PLA, Shijiazhuang, Hebei, China; 2 College of physical education, Huzhou normal university, Zhejiang, Huzhou, China; 3 School of Sports Science, Qufu Normal University, Qufu, Shandong, China; 4 Hebei University of Chinese Medicine, Shijiazhuang, Hebei, China; 5 School of Sport and Health Engineering, Hebei University of Engineering, Handan, Hebei, China

**Keywords:** anterior knee pain, blood flow restriction training, exercise therapy, muscle strength, patellofemoral pain syndrome

## Abstract

**Purpose:**

Previous studies suggest that blood flow restriction training (BFRT) may improve pain and knee function in individuals with patellofemoral pain syndrome (PFPS); however, its additional clinical benefit compared with general rehabilitation exercise (GRE) remains uncertain. This study aimed to compare the effects of BFRT and GRE in PFPS rehabilitation through a systematic review and meta-analysis.

**Methods:**

The Cochrane Library, PubMed, Web of Science, PEDro, Scopus, and ScienceDirect were searched up to February 14, 2026 (CRD420261296172). Interventional studies comparing BFRT with GRE in exercise-based rehabilitation for PFPS were included. Outcomes included pain intensity, self-reported knee function, knee extensor strength, and quadriceps morphology.

**Results:**

Thirteen studies were included qualitatively, and 11 randomized controlled trials involving 508 participants were included in the meta-analysis. BFRT produced immediate reductions in pain during functional tasks, including shallow single-leg squat (MD = 2.13; *P* < 0.00001), deep single-leg squat (MD = 2.38; *P* < 0.00001), and step-down tasks (MD = 2.05; *P* < 0.00001). This short-term analgesic effect remained significant 45 minutes after exercise. Over longer-term follow-up, BFRT showed a modest benefit for overall pain (SMD = 0.37; *P* = 0.0005) and knee extensor strength (SMD = 0.28; *P* = 0.01) compared with GRE. However, self-reported knee function did not differ significantly between groups (MD = 1.93; *P* = 0.12). Evidence for quadriceps morphology was limited, and no clear between-group difference was observed (SMD = 0.30; *P* = 0.24).

**Conclusion:**

In PFPS rehabilitation, BFRT may provide clinically relevant benefits for short-term pain reduction during functional tasks and modest improvements in knee extensor strength. However, current evidence does not show a clear advantage for self-reported knee function or quadriceps morphological outcomes. These findings should be interpreted cautiously because of protocol heterogeneity and limited long-term evidence.

**Systematic review registration**: https://www.crd.york.ac.uk/PROSPERO/view/CRD420261296172, identifier CRD420261296172.

patellofemoral pain syndrome (PFPS) is one of the most common clinical syndromes presenting as anterior knee pain. It is typically characterized by diffuse pain behind or around the patella, which is often aggravated during functional activities that increase patellofemoral joint loading, such as squatting, stair ascent or descent, and running (Ho et al., 2025). This condition is particularly prevalent among physically active populations, and its clinical course is frequently chronic or recurrent. Persistent pain and functional limitations can adversely affect sports participation, activities of daily living, and occupational performance, thereby reducing quality of life and imposing a measurable healthcare burden (Zambarano et al., 2022).
EG, experimental group; CG, control group; P, pneumatic; E, elastic; SBP, systolic blood pressure; LOP, limb occlusion pressure; RT, Resistance training; s/wk, sessions per week; NA, not applicable; NR, not reported; ①, pain intensity; ②, self-reported knee function score; ③, knee extensor strength; ④, quadriceps morphology; ⑤, physical performance.

The etiology of PFPS is multifactorial, and impaired knee extensor function and related neuromuscular control deficits have repeatedly been considered important contributing factors (Wallis et al., 2021). Clinically, reduced knee extensor strength and pain-related quadriceps inhibition may not only compromise performance in lower-limb load-bearing tasks but also limit the tolerable training load during rehabilitation. Consequently, individuals with PFPS may struggle to improve lower-limb strength, which in turn constrains functional recovery (Rethman et al., 2023). Therefore, PFPS rehabilitation should focus not only on pain reduction but also on restoring and enhancing knee extensor strength and related lower-limb physical capacity to support load tolerance and functional return.

Against this background, exercise therapy is widely regarded as a cornerstone of conservative management for PFPS. Traditional programs often recommend moderate-to-high load resistance training to improve knee extensor strength and thereby enhance load capacity during functional activities. However, in patients with high symptom irritability, during early-stage rehabilitation, or when functional limitations are prominent, high-load training may be difficult to implement or sustain due to symptom exacerbation, deteriorated movement quality, or reduced adherence. This may prevent consistent accumulation of an adequate training dose, ultimately leading to variable and sometimes suboptimal improvements in strength and function (Badiei et al., 2023). In other words, PFPS rehabilitation frequently faces a practical dilemma: sufficient mechanical stimulus is required, yet patients may be unable to tolerate sufficient external load at a given stage.

Blood flow restriction training (BFRT) has emerged as a potential load-management strategy, enabling resistance or rehabilitation exercises to be performed with lower external loads while aiming to improve knee-related muscle strength without markedly increasing joint loading, and potentially inducing quadriceps morphological adaptations (e.g., changes in muscle thickness or cross-sectional area) (Jørgensen et al., 2023). Moreover, the potential value of BFRT in PFPS may extend to improved exercise tolerance. Some trials have reported short-term reductions in pain provoked during standardized functional tasks; this transient hypoalgesia may create more feasible conditions for subsequent training, indirectly supporting completion of the intended training dose and progression of strength development (Sørensen et al., 2025b). Nevertheless, existing studies vary substantially in intervention formats and comparator conditions (e.g., high-load training, low-load non-occluded training, usual care, or placebo/sham compression). Reported outcomes for pain, function, and strength are also not fully consistent in direction and magnitude, making it difficult to derive clear, quantifiable prescription guidance for clinical practice.

Therefore, this study conducted a systematic review and meta-analysis to synthesize and evaluate the current evidence, focusing on core rehabilitation-relevant outcomes in PFPS, including pain intensity, self-reported knee function, knee extensor strength, and quadriceps morphology. We compared the effects of BFRT versus non-occluded training/usual rehabilitation on these outcomes in individuals with PFPS, quantified the intervention effects of BFRT, and aimed to provide a more robust evidence base to inform clinical training and rehabilitation decision-making, clarifying the potential role and practical value of BFRT within PFPS rehabilitation.

## Methods

### Protocol and registration

The study was conducted following the Preferred Reporting Items for Systematic Reviews and Meta-Analyses (PRISMA) statement checklist (Moher et al., 2009). Registration in the International Prospective Register of Systematic Reviews was made before starting, with the code CRD420261296172.

### Information sources and search

A Population, Intervention, Comparison, and Outcome (PICO) strategy was used to develop the search strategy. The population included adolescents and adults with PFPS or patellofemoral pain (PFP). The intervention comprised any blood flow restriction–related intervention involving lower-limb exercise or rehabilitation, including BFRT and other exercise-based approaches intended to restrict limb blood flow (e.g., intermittent blood flow restriction protocols). The comparison was sham or no blood flow restriction, usual care/physiotherapy, non-training control, and other exercise-based rehabilitation approaches (e.g., high-load or low-load resistance training without blood flow restriction). The outcomes included pain (including pain during standardized functional tasks), self-reported knee function, knee extensor strength, and quadriceps morphology.

Randomized controlled trial filters proposed by the Cochrane Collaboration were applied to the final search strategy. The search terms and keywords were organized according to the PICO framework and are presented in **Supplementary Appendix 1**. We searched PubMed, Web of Science, and Scopus, in addition to the Cochrane Central Register of Controlled Trials (CENTRAL), ScienceDirect, and PEDro. A manual search was also conducted, including screening the reference lists of included studies and relevant systematic reviews. The final search was performed on February 14, 2026. The complete search strategy for each database is provided in **Supplementary Appendix 2**.

### Outcomes

There were two main outcomes in this systematic review and meta-analysis. First, we assessed pain and self-reported knee function, focusing on patient-reported symptoms and perceived functional limitations related to patellofemoral pain. Pain outcomes included overall pain intensity and pain elicited during standardized functional tasks (e.g., single-leg squat and step-down tests). Self-reported knee function was evaluated using validated questionnaires [e.g., the Anterior Knee Pain Scale (AKPS)].

Second, we assessed objective knee-related and overall lower-limb physical function as secondary outcomes, defined as performance-based measures such as knee extensor strength and quadriceps morphological outcomes.

### Eligibility criteria

The inclusion criteria were as follows: (1) randomized controlled trial (RCT) study design; (2) adolescents or adults diagnosed with PFPS or PFP; (3) at least one intervention arm involving a blood flow restriction–related intervention applied in conjunction with lower-limb exercise or rehabilitation (e.g., BFRT or other exercise-based blood flow restriction approaches); (4) at least one relevant outcome reported, including pain (overall pain intensity or pain during standardized functional tasks), self-reported knee function (e.g., AKPS), knee extensor strength, and/or quadriceps morphology; and (5) sufficient quantitative data to calculate effect estimates (or data that could be derived from available statistics/figures).

The exclusion criteria were: (1) non-randomized designs (e.g., observational studies, case series, case reports); (2) studies primarily involving postoperative rehabilitation or acute traumatic knee injuries; (3) participants with knee pain predominantly attributable to other specific pathologies (e.g., advanced knee osteoarthritis, inflammatory arthritis, fracture, or other major structural knee disorders); and (4) interventions not intended to restrict limb blood flow or not combined with exercise/rehabilitation.

Two researchers independently screened titles, abstracts, and full texts. A third reviewer resolved disagreements. Interrater agreement was assessed using Cohen’s kappa statistic (Landis and Koch, 1977).

### Data extraction

For each included study, we extracted the following information: (1) first author’s last name; (2) year of publication; (3) study design and setting; (4) sample size and participant characteristics (e.g., age and sex distribution); (5) characteristics of the blood flow restriction–related intervention (intervention type; cuff/device type; occlusion/pressure prescription (e.g., %LOP or mmHg); exercise modality; training intensity and volume [sets and repetitions]; inter-set rest; session duration; intervention period; and training frequency); (6) comparator characteristics (e.g., sham/no blood flow restriction, usual care/physiotherapy, high-load or low-load resistance training, or non-training control); (7) outcomes and assessment details (measurement instruments, units, and timepoints); and (8) numerical outcome data and main results (means and standard deviations or other statistics required to calculate effect sizes).

Given the variability in BFRT protocols across the included studies, detailed intervention characteristics were extracted, including cuff/device type, cuff width, occlusion pressure prescription, exercise modality, training load, training volume, intervention frequency, and intervention duration. Quantitative synthesis was performed only when studies addressed the same clinical construct and involved exercise-based BFRT or blood-flow-restriction-related interventions in individuals with PFPS. To minimize clinical heterogeneity, acute single-session responses and longer-term intervention effects were analyzed separately. Accordingly, pooled estimates were interpreted as reflecting the average effect of BFRT-related exercise strategies rather than the effect of a single standardized BFRT prescription.

### Risk of bias

To assess the risk of bias of the randomized controlled trials included in this systematic review and meta-analysis, we used the Cochrane Risk of Bias tool 2.0 (RoB 2). RoB 2 evaluates risk of bias across five domains: (1) bias arising from the randomization process, (2) bias due to deviations from intended interventions, (3) bias due to missing outcome data, (4) bias in measurement of the outcome, and (5) bias in selection of the reported result. Following the RoB 2 signaling questions and algorithm, each domain was judged as “low risk,” “some concerns,” or “high risk,” and an overall risk-of-bias judgement was assigned for each study (and, where applicable, for each outcome). Two reviewers independently conducted the RoB 2 assessments. Disagreements were resolved through discussion, and, when necessary, a third reviewer adjudicated unresolved discrepancies. Risk of bias was assessed using the Cochrane RoB 2 tool, and risk-of-bias plots (traffic-light plots and summary bar plots) were generated using Robvis (McGuinness and Higgins, 2021).

### Statistical analyses

Meta-analyses were performed using Review Manager 5.3. For the primary analyses, change-from-baseline data were extracted for all continuous outcomes. When the mean change was not directly reported, it was calculated as the follow-up mean minus the baseline mean. When the standard deviation (SD) of the change score was not reported, it was derived from other available statistics (eg, standard errors, 95% confidence intervals, or *P* values), where possible. If these data were unavailable but both baseline and follow-up SDs were reported, the SD of the change score was estimated according to the Cochrane Handbook using the following formula:


$$SD_{\text{change}}=\sqrt{SD_{\text{baseline}}^{2}+SD_{\text{follow-up}}^{2}-2\times \text{Corr}\times SD_{\text{baseline}}\times SD_{\text{follow-up,}}}$$

where Corr denotes the correlation coefficient between baseline and follow-up measurements. Whenever possible, Corr was estimated from studies reporting baseline SD, follow-up SD, and change-score SD. When the required data were still unavailable, the corresponding authors were contacted to request the missing information.

When the same scale and units were used across studies, treatment effects were expressed as the mean difference (MD); when different instruments or units were used to assess the same construct, the standardized mean difference (SMD; Hedges’ g) was used. When studies reported mean change together with its standard error, the generic inverse variance (GIV) method was used to enter the corresponding effect estimates and their standard errors. For crossover trials, paired analyses were conducted using within-participant differences between conditions, and effect estimates together with their standard errors were entered using the generic inverse-variance method in RevMan.

Given the anticipated clinical and methodological heterogeneity across trials (eg, pressure prescription, training protocols, comparators, and assessment procedures), a random-effects model (DerSimonian–Laird method) was used as the primary pooling approach. Pooled effects were reported with 95% confidence intervals (CIs), and statistical significance was set at *P* < 0.05. Statistical heterogeneity was quantified using the I² statistic and interpreted as low (<25%), moderate (25%–75%), or high (>75%) (Higgins et al., 2003). To assess the robustness of the pooled estimates, leave-one-out sensitivity analyses were performed by removing each study in turn. When an outcome included ≥10 studies, funnel plots were visually inspected to explore potential publication bias; otherwise, publication bias was not formally assessed. To ensure a consistent direction of effect, outcomes were coded so that positive values favored the blood flow restriction intervention.

## Results

### Search strategy

The search strategy found 622 studies (Cochrane Library, n=172; PubMed, n=43; Web of Science, n=134; Pedro, n=94; Scopus, n=92; ScienceDirect, n=87). Two additional studies were included after the manual search. A total of 267 studies were initially considered to be included after removing duplicates.

### Study selection

**Figure 1:**
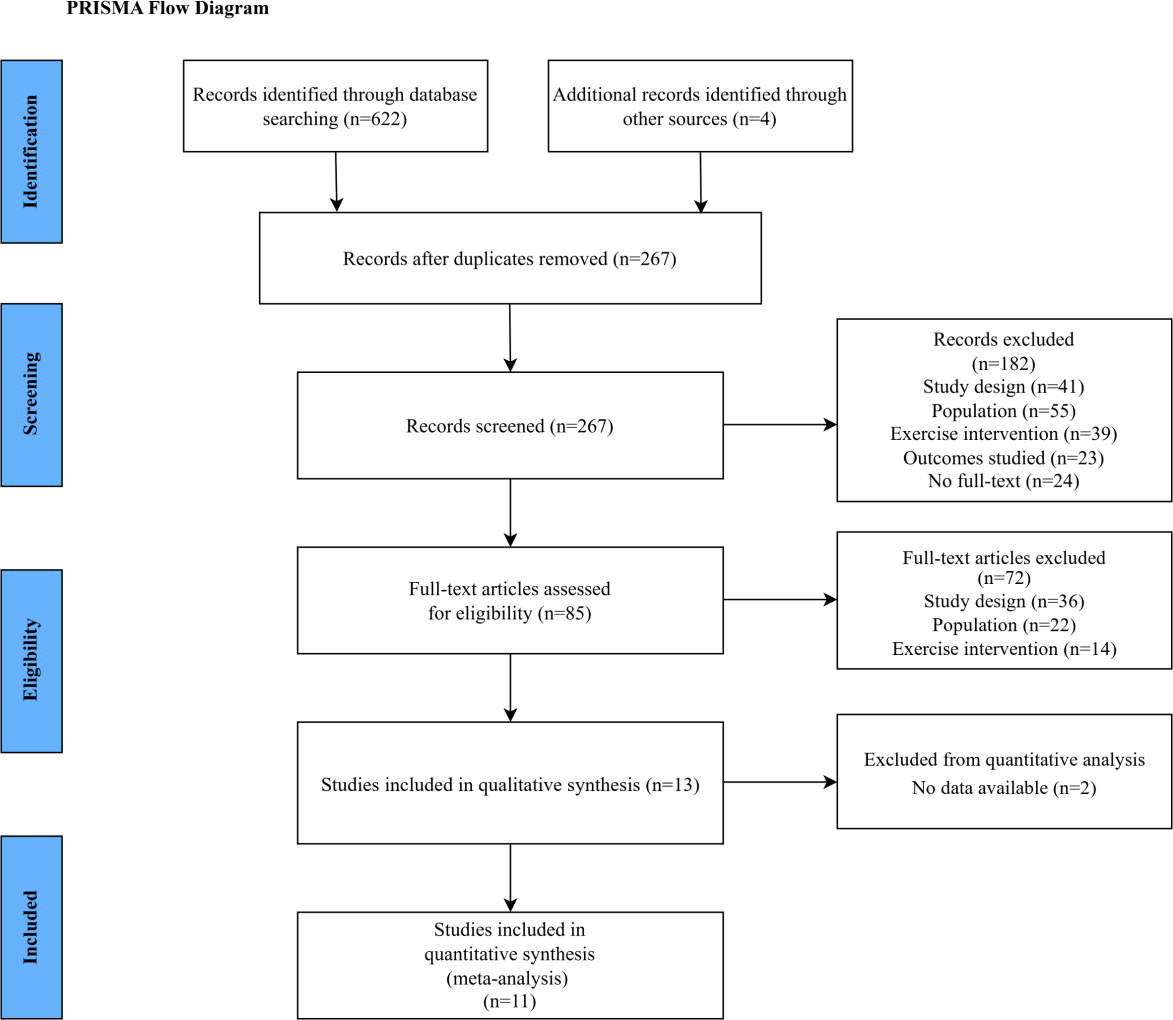
Search PRISMA flow diagram.

In the initial screening phase, after reviewing titles and abstracts, 182 records were excluded. Full texts were then retrieved for eligibility assessment, and 72 articles were excluded after full-text review. Ultimately, 13 randomized controlled trials met the inclusion criteria and were included in the qualitative synthesis, and 11 studies were included in the quantitative synthesis. Interobserver agreement for study selection was assessed using Cohen’s kappa (κ = 0.76), indicating substantial agreement (Landis and Koch, 1977). The PRISMA flow diagram is presented in **Figure 1**.

### Study characteristics

A total of 11 studies were included in the quantitative synthesis, comprising 508 participants, with sample sizes ranging from approximately 18 to 84 participants per study. Participants were predominantly adolescents and adults with PFPS or PFP, recruited from general clinical populations, recreationally active individuals, and specific occupational groups (e.g., military personnel). Regarding sex distribution, most studies included mixed male and female samples; three studies enrolled male-only samples (Korakakis et al., 2018b; Korakakis et al., 2018a; Al-Ogaili et al., 2025), one study enrolled a female-only sample (Girardi and Guenka, 2022), and one study did not clearly report the sex distribution in the abstract or main results.

Across included trials, intervention type and prescription varied. Overall, interventions could be categorized as cuff/device-based blood flow restriction and elastic band–based compression exercise interventions. Intervention duration ranged from single-session/short-term acute protocols to 4–9 weeks, with 8-week programs being most common. Training frequency was typically 2–3 sessions per week, although some studies used single-session or crossover designs. Interventions primarily consisted of low-load, single-joint resistance exercise combined with blood flow restriction (e.g., knee-focused resistance exercises such as leg press/leg extension), while some studies combined blood flow restriction with low-load, multi-joint exercises. Specific exercise selections are provided in **Supplementary Appendix 2**.

Comparators included sham/low-pressure placebo compression, the same training performed without blood flow restriction, usual care/physiotherapy, resistance training with different loading schemes (high-load or low-load), and non-intervention control conditions. The main outcomes encompassed pain intensity, self-reported knee function questionnaire scores, knee extensor strength, and quadriceps morphological measures; some studies also reported pressure pain threshold, performance-based outcomes, or other functional measures. Detailed characteristics of the included studies are summarized in **Table 1**.

**Table 1:** Characteristics of the studies included in the qualitative analysis.

Study	Population			EG				CG	Outcomes
	Total (EG/CG)	Sex,M/F	Age	Cuff type	Cuff width	Pressure, mmHg	Intervention protocol	Intervention protocol	
Lee et al. (2026)	39 (20/19)	23/16	28.05 ± 2.8	P	15cm	80% resting SBP	RT (load NR); 6 wk; 2 s/wk	RT (load NR); 6 wk; 2 s/wk	① ② ③ ④ ⑤
Kong et al. (2025)	42 (21/21)	27/15	19.0-21.0	P	10 cm	80% LOP	RT (30% 1RM); 8 wk; 3 s/wk	RT (70% 1RM) with sham cuff; 8 wk; 3 s/wk	① ② ③ ④
Al-Ogaili et al. (2025)	40	M	35.3 ± 11.4	P	10cm	80% LOP	Acute RT(0–5 kg)	Acute RT(0–5 kg)	①
León-Morillas et al. (2024)	50 (25/25)	14/36	46.58 ± 12	E	NR	60–80% LOP	RT(load NR); 8 wk; 2 s/wk	RT(load NR); 8 wk; 2 s/wk	① ② ③
Liu and Wu (2023)	26 (13/13)	16/10	20.92 ± 2.2	P	10cm	50 ± 8.0	RT (20%-50% 1RM); 4 wk; 2 s/wk	usual care	① ② ③
Talbot et al. (2023)	84 (42/42)	NR	28.5 ± 6.0	P	NR	80% LOP	RT (20-50% MVC); 9 wk; 2 s/wk	RT (20-50% MVC); 9 wk; 2 s/wk	① ③ ⑤
Constantinou et al. (2022)	60 (30/30)	33/27	30.5 ± 16	P	10cm	70% LOP	RT (30% 1RM); 4 wk; 3 s/wk	RT (70% 1RM); 4 wk; 3 s/wk	① ② ③ ④ ⑤
Girardi and Guenka (2022)	18 (9/9)	F	23.2 ± 1.98	P	6.5cm	143.33 ± 22(20+resting SBP)	RT (20% 1RM); 6 wk; 3 s/wk	RT (20% 1RM); 6 wk; 3 s/wk	① ② ③
Korakakis et al. (2018b)	40 (20/20)	M	29.1 ± 6.6	P	NR	80% LOP	Acute RT(0–5 kg)	Acute RT(0–5 kg)	①
Korakakis et al. (2018a)	30	M	29.7 ± 7.6	P	10cm	80% LOP	Acute RT(0–5 kg)	Acute RT(0–5 kg)	①
Giles et al. (2017)	79 (40/39)	43/36	28.5 ± 5.2	P	NR	60% LOP	RT (30% 1RM); 8 wk; 3 s/wk	RT (70% 1RM) with sham cuff; 8 wk; 3 s/wk	① ② ③ ④

EG, experimental group; CG, control group; P, pneumatic; E, elastic; SBP, systolic blood pressure; LOP, limb occlusion pressure; RT, Resistance training; s/wk, sessions per week; NA, not applicable; NR, not reported; ①, pain intensity; ②, self-reported knee function score; ③, knee extensor strength; ④, quadriceps morphology; ⑤, physical performance.

### Risk of bias assessment

**Figure 2:**
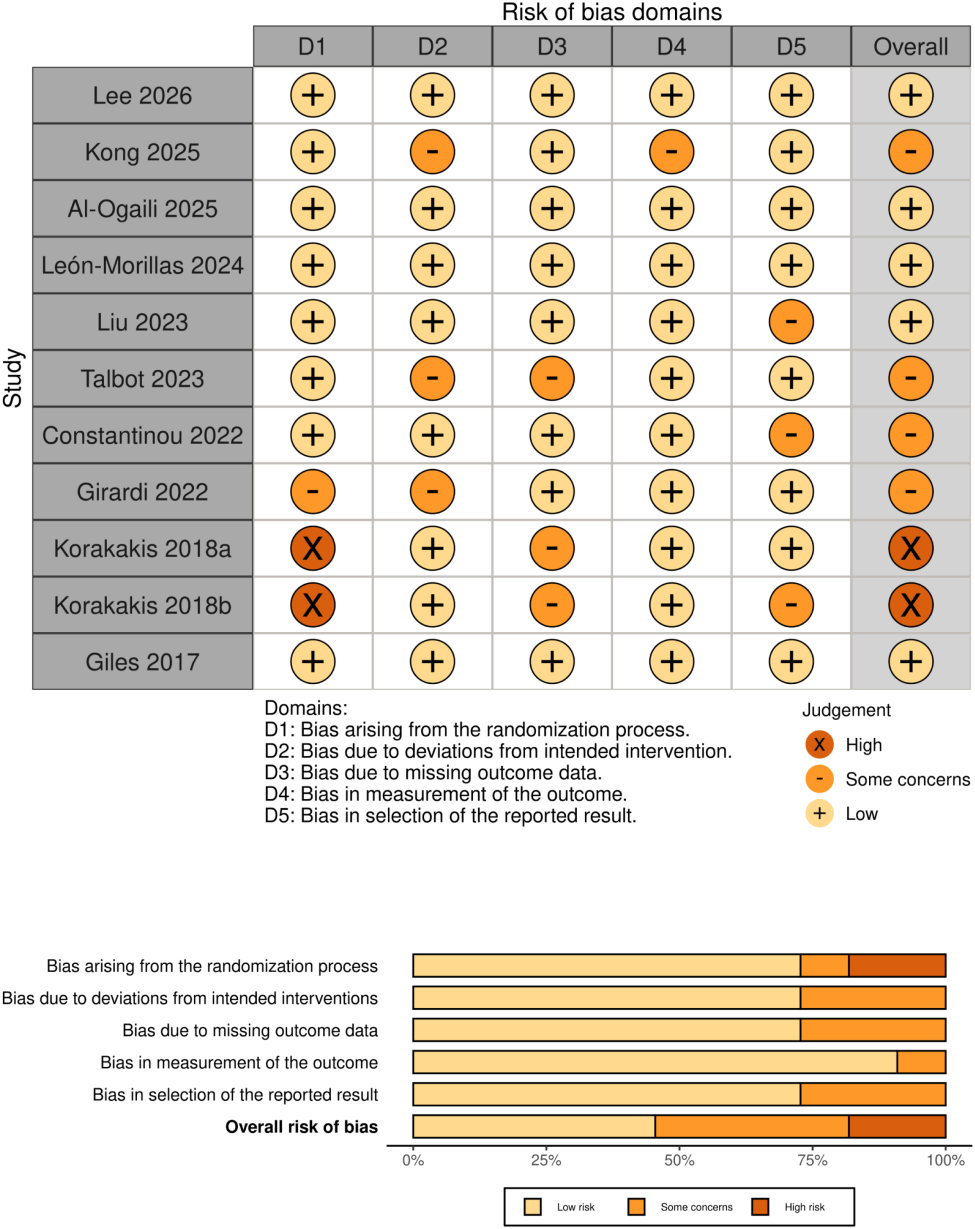
Risk of bias summary.

**Figure 2** shows the domain-level summary bar plot and the traffic-light plot generated with the RoB 2 tool and visualized using Robvis. Among the 11 studies included in the quantitative synthesis, seven studies were judged as having some concerns in at least one risk-of-bias domain, and two studies were judged as high risk in at least one domain. For the overall risk-of-bias judgement, five studies were rated as low risk, four as some concerns, and two as high risk.

## Outcomes

### Acute effects of BFRT on PFPS

Acute outcomes primarily assessed pain during functional tasks. Three studies (Korakakis et al., 2018b; Korakakis et al., 2018a; Al-Ogaili et al., 2025) (total n = 110) used a 0–10 Numerical Rating Scale (NRS) to rate pain intensity after standardized functional tasks, including the shallow single-leg squat, deep single-leg squat, and step-down test. Effect sizes were expressed as mean difference (MD) and subgrouped by assessment time point (immediate vs post-treatment [45 min after the intervention]). All pooled analyses used a random-effects model, and MD > 0 indicated greater pain reduction (baseline − post-test).

**Figure 3:**
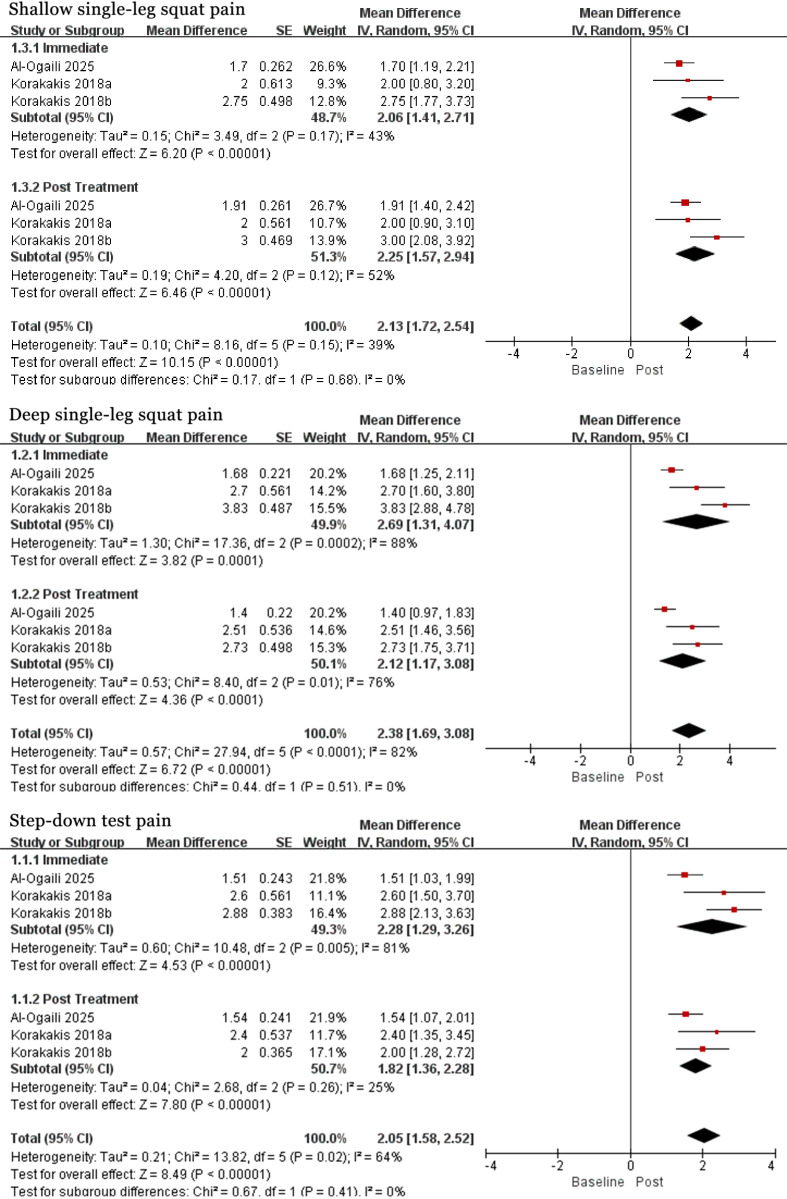
Effects of BFRT versus other interventions on pain during functional tasks.

For the shallow single-leg squat, the pooled immediate effect was MD = 2.06 (95% CI 1.41–2.71; I² = 43%; Z = 6.20, *P* < 0.00001), the post-treatment effect was MD = 2.25 (1.57–2.94; I² = 52%; Z = 6.46, *P* < 0.00001), and the overall pooled effect was MD = 2.13 (1.72–2.54; I² = 39%; Z = 10.15, *P* < 0.00001), with no significant subgroup difference between immediate and post-treatment effects (P = 0.68). For the deep single-leg squat, the immediate effect was MD = 2.69 (1.31–4.07; I² = 88%; Z = 3.82, *P* = 0.0001), the post-treatment effect was MD = 2.12 (1.17–3.08; I² = 76%; Z = 4.36, *P* < 0.0001), and the overall pooled effect was MD = 2.38 (1.69–3.08; I² = 82%; Z = 6.72, *P* < 0.00001), again with no significant subgroup difference (*P* = 0.51). For the step-down test, the immediate effect was MD = 2.28 (1.29–3.26; I² = 81%; Z = 4.53, *P* < 0.00001), the post-treatment effect was MD = 1.82 (1.36–2.28; I² = 25%; Z = 7.80, *P* < 0.00001), and the overall pooled effect was MD = 2.05 (1.58–2.52; I² = 64%; Z = 8.49, *P* < 0.00001), with no significant subgroup difference (*P* = 0.41)(**Figure 3**).

In addition, Al-Ogaili et al. (2025) reported other acute outcomes: compared with sham BFR, the experimental group showed greater improvement in subjective symptom ratings, a significant increase in knee pressure pain threshold, and significantly higher mean and peak ratings of perceived exertion (RPE).

### Long-term effects of BFRT on PFPS

#### Pain

A total of seven studies reported long-term pain intensity outcomes assessed using the VAS or NRS. Pain was categorized as worst pain and daily pain. Effect sizes were expressed as standardized mean difference (SMD) and subgrouped by worst pain versus daily pain. All pooled analyses used a random-effects model, and SMD > 0 indicated greater pain reduction.

**Figure 4:**
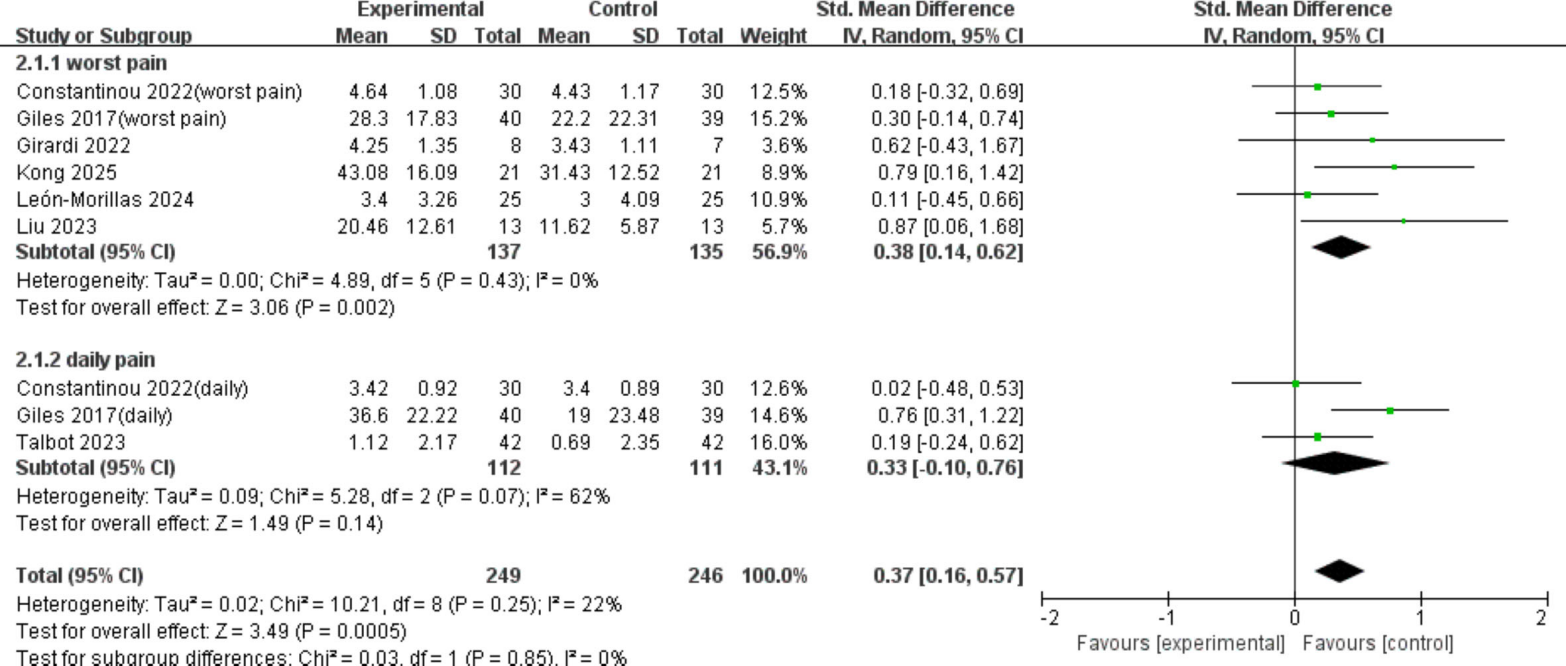
Effects of BFRT versus other interventions on pain.

The worst pain subgroup included six studies (experimental group n = 137; control group n = 135), yielding a pooled effect of SMD = 0.38 (95% CI 0.14–0.62; I² = 0%; Z = 3.06, *P* = 0.002). The daily pain subgroup included three studies (experimental group n = 112; control group n = 111), with a pooled effect of SMD = 0.33 (−0.10 to 0.76; I² = 62%; Z = 1.49, *P* = 0.14). The overall pooled effect was SMD = 0.37 (0.16–0.57; I² = 22%; Z = 3.49, *P* = 0.0005), and the between-subgroup difference was not significant (*P* = 0.85) (**Figure 4**).

### Self-reported knee function

**Figure 5:**
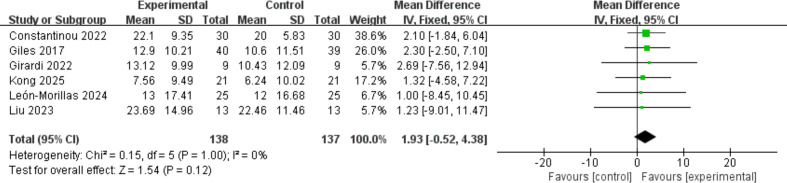
Effects of BFRT versus other interventions on self-reported knee function.

Six studies (Giles et al., 2017; Constantinou et al., 2022; Girardi and Guenka, 2022; Liu and Wu, 2023; León-Morillas et al., 2024; Kong et al., 2025) reported self-reported knee function scores, with a total sample size of n = 275 (experimental group n = 138; control group n = 137). A fixed-effect model was used for pooling, and MD > 0 indicated higher knee function scores. The overall pooled result showed no statistically significant between-group difference in improvements in self-reported knee function (MD = 1.93, 95% CI −0.52 to 4.38; Z = 1.54, *P* = 0.12). Between-study heterogeneity was low (Chi² = 0.15, df = 5, *P* = 1.00; I² = 0%) (**Figure 5**).

### Knee extensor strength

**Figure 6:**
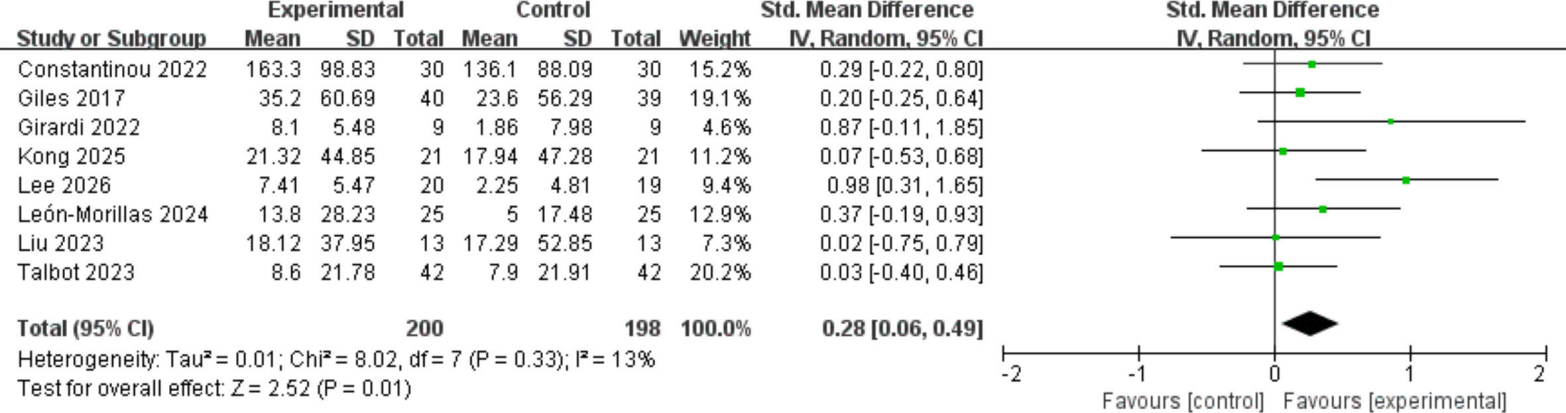
Effects of BFRT versus other interventions on knee extensor strength.

Knee extensor strength outcomes were used to assess pre–post changes in knee extensor muscle strength. Eight studies (total n = 398; experimental group n = 200; control group n = 198) reported this outcome. Knee extensor strength was primarily evaluated by measuring quadriceps maximal voluntary isometric contraction or peak torque. Effect sizes were pooled using the standardized mean difference (SMD), with SMD > 0 indicating greater gains in knee extensor strength in the experimental group. All pooled analyses used a random-effects model. The overall pooled effect was SMD = 0.28 (95% CI 0.06–0.49; Z = 2.52, *P* = 0.01), with low between-study heterogeneity (Chi² = 8.02, df = 7, *P* = 0.33; I² = 13%) (**Figure 6**).

### Quadriceps morphology

**Figure 7:**
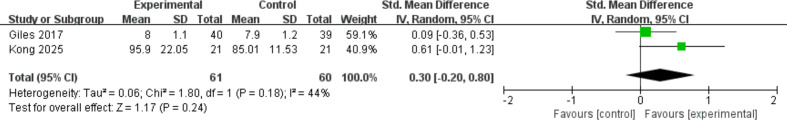
Effects of BFRT versus other interventions on quadriceps morphology.

For long-term outcomes, quadriceps morphology was primarily assessed using structural measures such as muscle cross-sectional area. Two studies reported this outcome, with a total sample size of n = 121 (experimental group n = 61; control group n = 60). Effect sizes were pooled using the standardized mean difference (SMD) with a random-effects model; SMD > 0 indicated greater improvement in quadriceps morphology in the experimental group. The pooled result suggested a small-to-moderate trend toward greater post-intervention improvements in quadriceps morphology in the experimental group compared with controls, but the difference did not reach statistical significance (SMD = 0.30, 95% CI −0.20 to 0.80; Z = 1.17, *P* = 0.24) (**Figure 7**).

### Other secondary outcomes

Three studies reported additional strength outcomes beyond knee extensor strength (Constantinou et al., 2022; Talbot et al., 2023; Lee et al., 2026). Constantinou et al. (2022) assessed maximal hip extensor and hip abductor strength using a handheld dynamometer. Both groups improved over time, but the group × time interaction was not significant (hip extension: *P* = 0.76; hip abduction: *P* = 0.71). Talbot et al. (2023)reported a non-significant time effect for involved-side knee flexor strength (*P* = 0.57); hip isometric strength also improved over time, but between-group effects were not significant. Lee et al. (2026) found that knee flexor isometric strength increased significantly from pre- to post-intervention in both groups (both *P* = 0.001), with no significant between-group difference (*P* = 0.359).

In addition, four studies assessed lower-limb functional performance tests (A G-LM et al., 2020; Ruas et al., 2022; Talbot et al., 2023; Lee et al., 2026). Talbot et al. (2023) reported improvements over time in the 30-s chair sit-to-stand, 30-s forward step-down, timed stair ascent/descent, and the 6-min walk test, but none of the group × time interactions were significant. Lee et al. (2026) reported improved Y-Balance scores in both groups (experimental group *P* = 0.015; control group *P* = 0.018; between-group difference *P* = 0.335), and greater improvement in the experimental group for the stair-descending functional task score (between-group difference *P* = 0.020). In the acute studies, Ruas et al. (2022) reported no significant differences between BFRT and non-occluded conditions for force-plate stability metrics during single-leg squatting (all *P* > 0.05). Marco et al. (2020) reported improved vertical jump performance following the BFRT, accompanied by an immediate reduction in knee pain (VAS 4.0 ± 0.7 to 0.5 ± 0.5; *P* < 0.001).

Only Lee et al. (2026) evaluated muscle mechanical properties using MyotonPRO. Both groups showed increases in lower-limb muscle tone and stiffness from pre- to post-intervention. In between-group comparisons, the experimental group demonstrated greater increases in muscle tone of the vastus medialis and vastus lateralis (*P* < 0.001 and *P* = 0.020, respectively), and greater increases in stiffness of the vastus medialis and semitendinosus (both *P* = 0.001), while between-group differences for other muscles were not significant (*P* > 0.05).

### Sensitivity analyses

Leave-one-out sensitivity analyses were conducted for each pooled outcome by sequentially removing one study at a time. The direction and statistical interpretation of the main findings were generally unchanged for pain intensity, self-reported knee function, knee extensor strength, and quadriceps morphology, indicating that the pooled results were not primarily driven by any single study. For acute pain outcomes, removal of Al-Ogaili et al (Al-Ogaili et al., 2025). substantially reduced heterogeneity, while the direction and significance of the pooled effects remained consistent (see **Supplementary Appendix 3** for details).

## Discussion

This systematic review and meta-analysis aimed to synthesize the evidence on the effects of BFRT–related lower-limb exercise interventions for PFPS, covering both acute (immediate responses following a single session) and long-term (outcomes after completion of an intervention period) dimensions. Existing international consensus statements and clinical guidelines for PFPS consistently emphasize exercise therapy as a core intervention to reduce pain and improve function. However, for BFRT as an “adjunctive” strategy, the evidence base remains uncertain. In this PFPS-specific synthesis, we integrated key outcomes—including pain intensity, self-reported function, and knee muscle strength—thereby providing more quantifiable information to support clinical decision-making and clarifying whether BFRT may serve as a valuable symptom-management approach when training loads need to be moderated in people with PFPS.

### Pain intensity

Prior evidence indicates that pain in PFPS is often distinctly load-related (e.g., pain is more readily provoked during squatting or stepping down). Accordingly, clinical practice commonly emphasizes completing exercise interventions that stimulate musculature without markedly exacerbating symptoms (Neal et al., 2024). Against this background, the potential value of BFRT is typically positioned as a load-management tool—maintaining a meaningful training/rehabilitation stimulus under relatively low external loads to achieve physiological effects similar to those of high-load training. It has also been noted that BFRT can be applied in low-load training contexts, but variability in prescription parameters (e.g., pressure settings and training modalities) may substantially influence physiological responses and intervention effects (Rolnick et al., 2023).

Our meta-analytic findings suggest that BFRT produces relatively consistent short-term improvements in pain provoked during functional tasks. Following a single BFRT session, pain ratings during shallow/deep single-leg squats and step-down tasks decreased by approximately two points both immediately and at 45 minutes post-treatment, with no significant subgroup difference between time points. This magnitude of reduction may be clinically meaningful: in PFPS populations, the minimal clinically important difference (MCID) for NPRS has been estimated at a reduction of approximately 1.16 points, and the clinically important difference for a 10-cm VAS is often reported to be around 2 cm (Piva et al., 2009). These findings may help explain why BFRT could be more feasible for individuals with PFPS—exercise can be performed on the basis of lower pain, potentially reducing discomfort during training. Nonetheless, it is important to note that, in the included studies, the BFRT group commonly reported higher ratings of perceived exertion and greater post-exercise muscle soreness, suggesting that some individuals with PFPS may overexert during BFRT. This could negatively affect patient experience and adherence, thereby attenuating potential benefits (de Queiros et al., 2023). From a methodological perspective, heterogeneity (I²) was higher for deep single-leg squat and step-down outcomes, whereas it was relatively lower for the shallow single-leg squat. Heterogeneity decreased markedly in leave-one-out analyses while conclusions remained consistent, suggesting that heterogeneity was more likely attributable to differences in task standardization, baseline pain levels, and related factors rather than contradictory directions of effect.

Regarding long-term effects, pooled estimates of pain intensity further support the notion that BFRT-related benefits may be more concentrated in pain improvement during higher-load functional contexts. Subgroup analyses showed that worst pain demonstrated a stable small-to-moderate effect with virtually no heterogeneity (SMD = 0.38; I² = 0%), whereas daily pain showed a smaller, non-significant effect with moderate heterogeneity (SMD = 0.33; I² = 62%). The overall pooled effect indicated a small-to-moderate improvement (SMD = 0.37; I² = 22%). “Worst pain” often reflects pain experienced during a high-load knee task within a given week and may be more sensitive to improvements in load tolerance achieved through training (Karanasios et al., 2023). As patients gradually perform previously painful functional tasks with less or no pain, worst pain may decrease relatively quickly. In contrast, “daily pain” represents an averaged description of pain over a day or period of time and is more susceptible to fluctuations in daily activity volume, training scheduling, and measurement timing (Xiong et al., 2025). Moreover, most interventions in this review lasted only 4–9 weeks. Within such a limited timeframe, improvements may be more likely to emerge first as reductions in peak pain during high-load tasks, whereas changes in average daily pain may require longer interventions.

### Self-reported knee function

Self-reported knee function is one of the core patient-centered outcomes in PFPS research, reflecting pain-related limitations in activities of daily living and overall functional recovery. In the included studies, self-reported knee function was most commonly assessed using the AKPS (Crossley et al., 2004). Previous work indicates that the AKPS can discriminate between individuals who have “improved” versus “not improved” in PFPS populations, and that the minimal important difference perceived by patients is typically around 10 points. The minimal important change (MIC) for the AKPS has also been estimated at approximately 11 points (Hott et al., 2021). In our quantitative synthesis of long-term interventions, BFRT showed only a trend toward improvement compared with controls, without a statistically significant advantage. This aligns with related evidence; for example, Giles et al. reported that, in a double-blind randomized trial comparing low-load BFRT with conventional quadriceps training, the between-group difference in AKPS was likewise not significant, and no divergence between groups was observed during follow-up.

From a clinical perspective, the pooled estimate in this review (MD ≈ 2 points) is clearly below the commonly cited MIC threshold for the AKPS in PFPS. This suggests that even if BFRT confers a true advantage in function scores, the magnitude is unlikely to be large enough to produce a consistent, perceptible additional benefit at the patient level. Therefore, current evidence more strongly supports BFRT as an alternative training option during phases when pain sensitivity is high or high-load exercise is poorly tolerated—potentially enabling patients to complete training at lower external loads—rather than as a strategy that outperforms high-quality conventional training or other comparators in improving self-reported knee function.

It is also important to note that exercise therapy itself yields clear benefits for patient-reported outcomes in PFPS (pain and functional activities). When control groups similarly receive structured exercise or comprehensive rehabilitation, the incremental effect of adding a single “adjunct component” is often limited and between-group differences can be diluted. In addition, the AKPS may exhibit some ceiling effects and only moderate responsiveness in PFPS populations, which may further reduce the likelihood of detecting short-duration between-group differences. Consistent with this, other meta-analyses have shown that, compared with exercise alone, combined interventions for PFPS often yield only small and non-significant between-group differences in Kujala scores, indirectly suggesting that functional improvement in PFPS may depend more on the overall quality of exercise delivery and adherence than on any single technique (Abdelhamed et al., 2025).

### Knee extensor strength and quadriceps morphology

The meta-analysis in this review showed that BFRT produced a small but statistically significant improvement in knee extensor strength (SMD = 0.28, 95% CI 0.06–0.49). This suggests that, in people with PFPS, despite heterogeneity across studies in exercise selection, occlusion/pressure prescription, and comparator conditions, strength outcomes still demonstrated a relatively consistent direction of benefit. Knee extensor strength is determined not only by intrinsic muscle capacity but also by pain-related inhibition (Sørensen et al., 2025a). Pain associated with PFPS can reduce voluntary quadriceps activation, making strength test results susceptible to symptom fluctuations. When an intervention reduces pain in the short term and improves movement tolerance and participation, strength test performance may increase via improved neuromuscular recruitment even before meaningful structural muscle changes occur (Badiei et al., 2023). In addition, by inducing greater metabolic stress at relatively low external loads, BFRT typically allows individuals to achieve higher training volume and/or a stimulus closer to fatigue under low-load conditions, providing a plausible training-based rationale for strength gains.

Against this background, changes in quadriceps morphology may be considered a potential explanatory factor for improvements in knee extensor strength. However, the present review cannot confirm—based on morphological evidence—that the observed strength gains were primarily driven by structural hypertrophy. Only two studies reported quadriceps morphological outcomes, and the pooled estimate suggested a small-to-moderate trend toward improvement that did not reach statistical significance (SMD = 0.30, 95% CI −0.20 to 0.80), reflecting insufficient sample size and limited study numbers.

Several reasons may account for the non-significant morphological findings. First, most interventions were relatively short in duration, and structural adaptations typically lag behind neuromuscular adaptations. Second, most control groups also received effective exercise-based training, making it more difficult to “separate” between-group differences in structural outcomes. This is also consistent with broader BFRT meta-analytic evidence: in general, or other clinical populations, prior meta-analyses often report that low-load BFRT can induce hypertrophy comparable to high-load resistance training, but such conclusions are usually supported by larger samples and more standardized comparator interventions. In PFPS—where the evidence base remains limited—structural evidence is still at an early stage of accumulation. The evidence regarding quadriceps morphology therefore remains insufficient. Only a limited number of studies reported morphological outcomes, and the pooled result did not show a clear between-group effect. In addition, differences in measurement methods, intervention duration, and BFRT prescription may have contributed to uncertainty. Further adequately powered randomized controlled trials using standardized imaging-based assessments and longer follow-up periods are required to determine whether BFRT can induce meaningful quadriceps morphological adaptations in individuals with PFPS.

From a practical perspective, these results support viewing BFRT as an adjunct within PFPS exercise prescription rather than a fixed first-line option. Specifically, when patients have poor tolerance to high-load training early in rehabilitation, or when pain during leg extension, squatting, or similar tasks limits the achievable training dose, BFRT may enable sufficient training stimulus under lower external loads, thereby helping maintain training continuity and promoting increases in knee extensor strength (Jacobs et al., 2025). In practice, BFRT may be best integrated into a progressive loading program: low-load BFRT can be used within symptom-tolerated ranges initially, followed by a gradual transition toward conventional training (increasing external load and reducing reliance on BFRT) as pain sensitivity decreases and load tolerance improves, to achieve the overarching goals of long-term load management and recovery of physical capacity. Overall, this review suggests that BFRT offers a reproducible, small advantage for strength gains, whereas morphological evidence remains insufficient; thus, in current practice it may be most appropriately considered a tool to help patients overcome phase-specific “training bottlenecks.”

### Clinical implications

The findings of this review suggest that BFRT may be considered as an adjunctive load-management strategy within PFPS/PFP rehabilitation rather than as a replacement for guideline-recommended exercise therapy. In clinical practice, BFRT may be most relevant for patients who have difficulty tolerating moderate- or high-load strengthening because of pain, symptom irritability, or reduced load tolerance. Low-load BFRT may be used during the early or symptom-sensitive phase of rehabilitation to help patients complete strengthening exercises with lower external loads while maintaining a sufficient training stimulus. As pain decreases and load tolerance improves, rehabilitation should progressively transition toward conventional hip- and knee-focused strengthening, functional loading, and sport- or activity-specific exercise. Because current evidence does not show a clear additional benefit for self-reported knee function and remains insufficient for quadriceps morphology, BFRT should be applied cautiously as part of a broader progressive rehabilitation program, with individualized cuff prescription, symptom monitoring, and attention to patient tolerance.

### Study limitations

Several limitations of this systematic review and meta-analysis should be acknowledged. First, the included trials exhibited clinical and methodological heterogeneity. Intervention duration ranged from single-session acute protocols to 4–9 weeks, and intervention types included both pneumatic cuff/device-based BFRT and elastic band–based compression approaches. Comparator conditions were likewise diverse, including sham/low-pressure placebo compression, the same training performed without BFRT, usual care/physiotherapy or rehabilitation, resistance training with different loading schemes (high-load or low-load), and non-intervention controls. In addition, BFRT prescription parameters varied substantially across studies, such as the pressure prescription method and level (e.g., %LOP vs fixed pressure), cuff/device type, and cuff width. These differences may jointly influence the physiological stimulus, tolerability, and ultimately the clinical effects of the interventions, thereby reducing comparability and generalizability.

Second, between-group comparisons in some studies did not constitute strict dose-matched analyses. The primary contrast was often “with versus without BFRT,” but the intervention and control groups could also differ in exercise content, total training dose (sets × repetitions × load), progression strategies, or co-interventions (e.g., components of usual rehabilitation). Therefore, observed between-group differences—particularly for strength- and performance-related outcomes—may partly reflect differences in overall training dose/content rather than the isolated effect of BFRT per se.

Third, variability in outcome measurement methods and limited evidence volume for some outcomes constrained the strength of inference. Although knee extensor strength was reported in more studies, testing procedures were not fully consistent (e.g., maximal voluntary isometric contraction vs peak torque). Quadriceps morphology was reported in only two studies, resulting in wider confidence intervals and limited statistical power, precluding definitive conclusions regarding structural adaptations. Moreover, for most outcomes the number of included studies did not reach ≥10, which precluded formal assessment of publication bias and limited the feasibility of reliable subgroup analyses (e.g., by comparator type, prescription parameters, or intervention duration) to explore sources of heterogeneity.

## Conclusions

Current evidence suggests that BFRT may provide clinically relevant short-term pain relief during functional tasks and modest improvements in knee extensor strength in individuals with PFPS. However, BFRT did not demonstrate a clear advantage over general rehabilitation exercise for self-reported knee function, and the evidence for quadriceps morphological adaptations remains insufficient. Given the heterogeneity of BFRT protocols, variability in cuff prescription, risk-of-bias concerns, and limited long-term follow-up data, these findings should be interpreted cautiously. BFRT should therefore be considered a potential adjunctive load-management strategy for patients who have difficulty tolerating higher-load exercise, rather than a replacement for guideline-recommended progressive hip- and knee-focused exercise rehabilitation. Further high-quality trials with standardized BFRT prescription, adequate follow-up, and clinically relevant outcomes are needed to clarify its long-term effectiveness and optimal role in PFPS rehabilitation.

## Data Availability

The data analyzed in this study were derived from previously published studies. All extracted data supporting the findings of this systematic review and meta-analysis are included in the article and/or **Supplementary Material**. Further inquiries can be directed to the corresponding author.
