# Supplementary material for: Effects of blood flow restriction exercise interventions on patellofemoral pain syndrome: a systematic review and meta-analysis
Source: Front Physiol. 2026 Jun 18;17:1859305. doi: 10.3389/fphys.2026.1859305 (PMC13322932; doi:10.3389/fphys.2026.1859305)
Supplement: Supplementary file 1 [file Table1.docx]

**Appendix 1. Search Terms and Keywords Classified According to the PICO Framework**

**Population（PFP/PFPS）**

- **MeSH（PubMed）**
  - “Patellofemoral Pain Syndrome”
- **Keywords（Title/Abstract）**
  - patellofemoral pain
  - patellofemoral pain syndrome
  - PFPS OR PFP
  - anterior knee pain
  - runner* knee
  - retropatellar pain
  - patellofemoral syndrome
  - patellar pain

**Intervention（blood flow restriction–related interventions）**

- **Keywords（Title/Abstract）**
  - blood flow restriction OR blood-flow restriction
  - BFR OR BFRT
  - KAATSU OR kaatsu training
  - occlusion training
  - vascular occlusion OR blood flow occlusion
  - venous occlusion
  - tourniquet* OR cuff*
  - intermittent blood flow restriction OR iBFR
  - tissue flossing OR flossing
  - floss band OR flossing band OR compression band OR elastic band*

**Comparison**

- sham OR placebo OR control
- usual care OR physiotherapy OR rehabilitation
- resistance training OR strength training
- high load OR low load
- no training OR waitlist

**Outcomes**

- pain OR VAS OR NRS OR NPRS
- function OR knee function
- AKPS OR “Anterior Knee Pain Scale” OR Kujala
- Lysholm OR LEFS
- strength OR torque OR 1RM OR dynamomet* OR isokinetic
- quadriceps OR morphology OR thickness OR “cross-sectional area” OR CSA OR ultrasound

**Search strategies (final search: February 14, 2026)**

( "Patellofemoral Pain Syndrome"[Mesh] OR "patellofemoral pain"[tiab] OR "patellofemoral pain syndrome"[tiab] OR PFPS[tiab] OR PFP[tiab] OR "anterior knee pain"[tiab] OR "runner* knee"[tiab] OR retropatellar[tiab])

AND

("blood flow restriction"[tiab] OR "blood-flow restriction"[tiab] OR BFR[tiab] OR BFRT[tiab] OR KAATSU[tiab] OR "kaatsu training"[tiab] OR "occlusion training"[tiab] OR "vascular occlusion"[tiab] OR "blood flow occlusion"[tiab] OR "venous occlusion"[tiab] OR tourniquet*[tiab] OR cuff*[tiab] OR "intermittent blood flow restriction"[tiab] OR iBFR[tiab] OR "tissue flossing"[tiab] OR flossing[tiab] OR "floss band"[tiab] OR "flossing band"[tiab])
