# Supplementary material for: Effects of blood flow restriction exercise interventions on patellofemoral pain syndrome: a systematic review and meta-analysis
Source: Front Physiol. 2026 Jun 18;17:1859305. doi: 10.3389/fphys.2026.1859305 (PMC13322932; doi:10.3389/fphys.2026.1859305)
Supplement: Supplementary file 2 [file Table2.docx]

**Appendix 2. Summary of Exercise and Training Content in the Included Studies (n = 13)**

Table A2.1. Exercise and training content reported in the included studies

| **Study** | **Exercises (abbrev.)** | **Exercises (description)** |
| --- | --- | --- |
| Giles 2017 | LP, LE | Leg press; leg extension. |
| Constantinou 2022 | HAb, HExt, HER, LE, LC, SQ, SLP | Hip and knee strengthening program incl. hip abduction/extension/external rotation; seated knee extension; prone knee flexion; squatting; single-leg press. |
| Kong 2025 | LP, LE | Leg press (0-60°); seated knee extension (90-45°). |
| Girardi 2022 | LP, LE | Leg extension machine; leg press. |
| Lee 2026 | LE, HAb, HER, SQ | Knee extension; hip abduction; hip external rotation; squat. |
| Liu 2023 | LC, HFSQ | Leg curl (seated/standing); weighted hip flexion squat. |
| Talbot 2023 | TherEx (STR + FUNC) | Therapeutic exercise program (strengthening + functional drills) delivered alongside NMES (and BFR/sham cuff depending on group). |
| Korakakis 2018a | LE (OKC) | Open kinetic chain knee extension (single session). |
| Korakakis 2018b | LE (OKC) | Open kinetic chain knee extension (single session; no control group). |
| Al-Ogaili 2025 | LE (OKC) | Open kinetic chain knee extension (single session; crossover: high vs low occlusion pressure). |
| Ruas 2022 | SLSQ | Single-leg squat task performed on a force platform (with/without partial vascular occlusion). |
| Marco 2020 | TF + CMJ | Tissue flossing applied around the knee; countermovement jump testing (acute). |
| León-Morillas 2024 | STR (Q/Ham/HAb) + PRO | Strengthening of quadriceps/hamstrings/hip abductors plus proprioception (e.g., single-leg stance, controlled knee flexion); flossing band added in the experimental arm. |

**Abbreviations:**

| **Abbrev.** | **Meaning** |
| --- | --- |
| CMJ | countermovement jump |
| FUNC | functional drills |
| HAb | hip abduction |
| HExt | hip extension |
| HER | hip external rotation |
| HFSQ | weighted hip flexion squat (as named in the study) |
| LC | leg curl / knee flexion exercise |
| LE | leg extension / knee extension |
| LP | leg press |
| OKC | open kinetic chain |
| PRO | proprioception/balance training |
| SLP | single-leg press |
| SLSQ | single-leg squat |
| SQ | squat |
| STR | strengthening |
| TF | tissue flossing |
| TherEx | therapeutic exercise |
