# Supplementary material for: Effects of blood flow restriction exercise interventions on patellofemoral pain syndrome: a systematic review and meta-analysis
Source: Front Physiol. 2026 Jun 18;17:1859305. doi: 10.3389/fphys.2026.1859305 (PMC13322932; doi:10.3389/fphys.2026.1859305)
Supplement: Supplementary file 3 [file Table3.docx]

**Appendix 3. Leave-one-out sensitivity analyses**


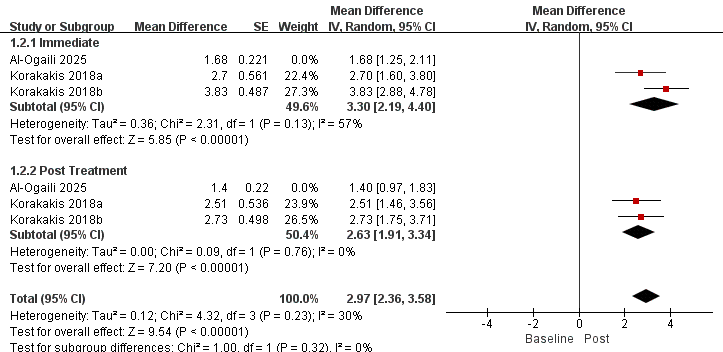


Figure A3.1. Sensitivity analysis of pain during the shallow single-leg squat


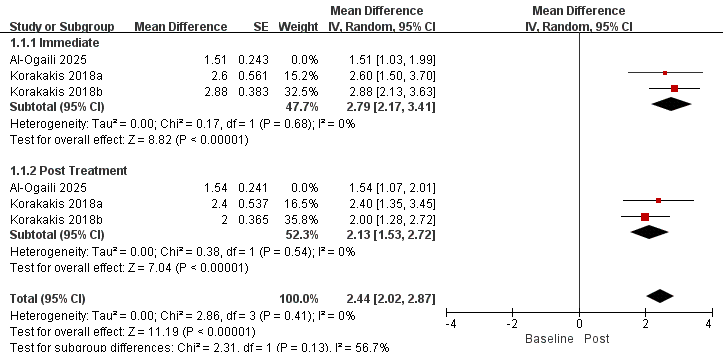


Figure A3.2. Sensitivity analysis of pain during the deep single-leg squat


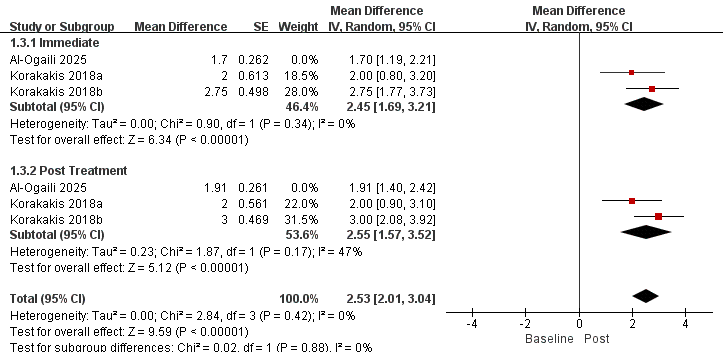


Figure A3.3. Sensitivity analysis of pain during the step-down test
